# Using epigenetic clocks to investigate changes in the age structure of critically endangered Māui dolphins

**DOI:** 10.1002/ece3.10562

**Published:** 2023-09-28

**Authors:** Keith M. Hernandez, Kaimyn B. O'Neill, Eleanor K. Bors, Debbie Steel, Joseph A. Zoller, Rochelle Constantine, Steve Horvath, C. Scott Baker

**Affiliations:** ^1^ Marine Mammal Institute Oregon State University Oregon Newport USA; ^2^ Fielding School of Public Health, Department of Biostatistics University of California California Los Angeles USA; ^3^ School of Biological Sciences & Institute of Marine Science University of Auckland – Waipapa Taumata Rau Auckland New Zealand; ^4^ David Geffen School of Medicine, Department of Human Genetics University of California California Los Angeles USA; ^5^ Altos Labs California San Diego USA

**Keywords:** age structure, *Cephalorhynchus* dolphins, DNA methylation, epigenetics, molecular aging, population dynamics

## Abstract

The age of an individual is an essential demographic parameter but is difficult to estimate without long‐term monitoring or invasive sampling. Epigenetic approaches are increasingly used to age organisms, including nonmodel organisms such as cetaceans. Māui dolphins (*Cephalorhynchus hectori maui*) are a critically endangered subspecies endemic to Aotearoa New Zealand, and the age structure of this population is important for informing conservation. Here we present an epigenetic clock for aging Māui and Hector's dolphins (*C. h. hectori*) developed from methylation data using DNA from tooth aged individuals (*n* = 48). Based on this training data set, the optimal model required only eight methylation sites, provided an age correlation of .95, and had a median absolute age error of 1.54 years. A leave‐one‐out cross‐validation analysis with the same parameters resulted in an age correlation of .87 and median absolute age error of 2.09 years. To improve age estimation, we included previously published beluga whale (*Delphinapterus leucas*) data to develop a joint beluga/dolphin clock, resulting in a clock with comparable performance and improved estimation of older individuals. Application of the models to DNA from skin biopsy samples of living Māui dolphins revealed a shift from a median age of 8–9 years to a younger population aged 7–8 years 10 years later. These models could be applied to other dolphin species and demonstrate the ability to construct a clock even when the number of known age samples is limited, removing this impediment to estimating demographic parameters vital to the conservation of critically endangered species.

## Graphical abstract

We developed epigenetic clocks to age critically endangered Maui dolphins based on DNA methylation data from the skin. Additionally, we developed a beluga/dolphin clock to improve age estimation for older individuals. We found a shift in the age distribution of Maui dolphins to an overall younger median age.

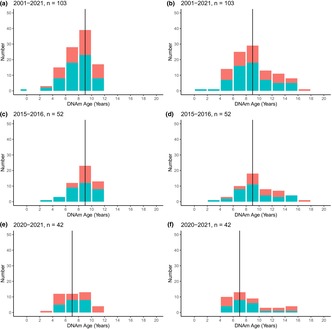

## 1 INTRODUCTION

Determining the ages of individuals is critical for understanding the dynamics of a population and for informing effective conservation and management actions. However, age is difficult to assess in wild animal populations, particularly those with life spans longer than typical field efforts. Additionally, age can rarely be determined by appearance and instead requires samples obtained invasively or lethally. This is counterproductive for species of conservation concern. For toothed whales (dolphins and porpoises) age is typically estimated by counting growth layer groups in teeth extracted from a living or dead individual (Bowen & Northridge, 2010). The technique is not without biases though, as tooth wear can lead to underestimation of chronological age (Bowen & Northridge, 2010; Hohn, & Fernandez, 1999). Age can also be inferred from sighting histories of naturally marked individuals, but this is only possible for well‐observed populations in coastal habitats (Hammond et al., 2021). Thus, minimally invasive approaches to estimating age are needed for most wild populations of cetaceans, providing a valuable addition to the ongoing development of molecular tools critical for informing conservation management (Formenti et al., 2022; Hohenlohe et al., 2021; Parrott & Bertucci, 2019).

Molecular aging is now largely based on DNA methylation (De Paoli‐Iseppi et al., 2017; Horvath, 2013; Horvath & Raj, 2018). DNA methylation refers to the addition of methyl groups (–CH_3_) throughout the genome, typically at cytosine‐guanine dinucleotides (CpGs). The ratio of methylation to nonmethylation at these CpG sites can be positively or negatively correlated with the chronological age of individuals in species ranging from mice to humans (e.g., Horvath, 2013; Thompson et al., 2018), including nonmodel organisms such as cetaceans (Beal et al., 2019; Bors et al., 2021; Peters et al., 2022; Robeck, Fei, Haghani, et al., 2021a). Multi‐species clocks are also developed to examine trends in aging across taxa (e.g., Parsons et al., 2023; Robeck, Fei, Haghani, et al., 2021a). Penalized linear regression models have been constructed from CpG sites to develop “epigenetic clocks,” relating percent methylation at informative sites to derive an estimate of age (DNAm, Horvath, 2013, Horvath & Raj, 2018). Alternatively, age can be estimated based on methylation at a small number of targeted CpG sites, as opposed to surveying the genome with bead‐based arrays (e.g., Beal et al., 2019; Polanowski et al., 2014). Given that clock models can be applied to small samples of skin or blood collected during routine field efforts, there is considerable promise for developing minimally invasive estimates of ages for populations of conservation concern.

Hector's and Māui dolphins (*Cephalorhynchus hectori hectori* [Van Bénéden 1881]) and (*C. h. maui* [Baker et al., 2002]) are endemic to the coastal waters of Aotearoa New Zealand. The Māui dolphin is the world's rarest marine dolphin, classified as critically endangered by the International Union for the Conservation of Nature (IUCN) and nationally critical in the New Zealand Threat Classification System (Baker et al., 2019; Reeves et al., 2013). Fisheries closures have provided greater protection from the long‐standing threat of bycatch (Department of Conservation & Fisheries New Zealand, 2021). A newer concern is the threat of disease associated with increasing prevalence of *Toxoplasma gondii* (Constantine & Baker, 2022; Roberts et al., 2021; Roe et al., 2013). Recent boat‐based and genetic capture‐recapture surveys have estimated the current population size of Māui dolphins at 54 individuals aged 1 year or older, and an effective population size (N_e_) of 35 (Constantine et al., 2021). Skin biopsy samples collected during routine survey efforts, as well as samples from dead beachcast and bycaught dolphins have resulted in a growing database of individually genotyped dolphins, and an archive of extracted DNA to test in the development of an epigenetic clock.

Here, we developed and validated two sets of epigenetic clocks for Māui and Hector's dolphins based on DNA methylation data and a training data set of individuals aged by teeth growth layer groups. We also leveraged preexisting DNA methylation training data from skin collected from beluga whales (*Delphinapterus leucas*, Pallas 1776) to develop a joint beluga/dolphin clock, contributing to similar multi‐species clocks for cetaceans (Parsons et al., 2023; Robeck, Fei, Lu, et al., 2021b). We then applied the clocks to generate DNAm age estimates from biopsy samples of living Māui dolphins to determine the age structure of the population during two survey periods. Based on observations during boat‐based surveys and genetic capture–recapture data collection (Constantine et al., 2021), we hypothesize that the population of Māui dolphins has shifted to a younger median age over recent survey years. The models developed here advance our understanding of epigenetic clocks for aging cetaceans, particularly in the context of contributing to improved conservation and management (Beal et al., 2019; Bors et al., 2021; Horvath et al., 2022; Peters et al., 2022; Polanowski et al., 2014; Robeck, Fei, Haghani, et al., 2021a). Critically, this approach shows promise for the development and application of epigenetic clocks from species that are elusive or logistically challenging to study, and those with small population sizes, which may not meet suggested sampling protocols (e.g., Mayne et al., 2021).

## 2 METHODS

### 2.1 Sample collection

**FIGURE 1:**
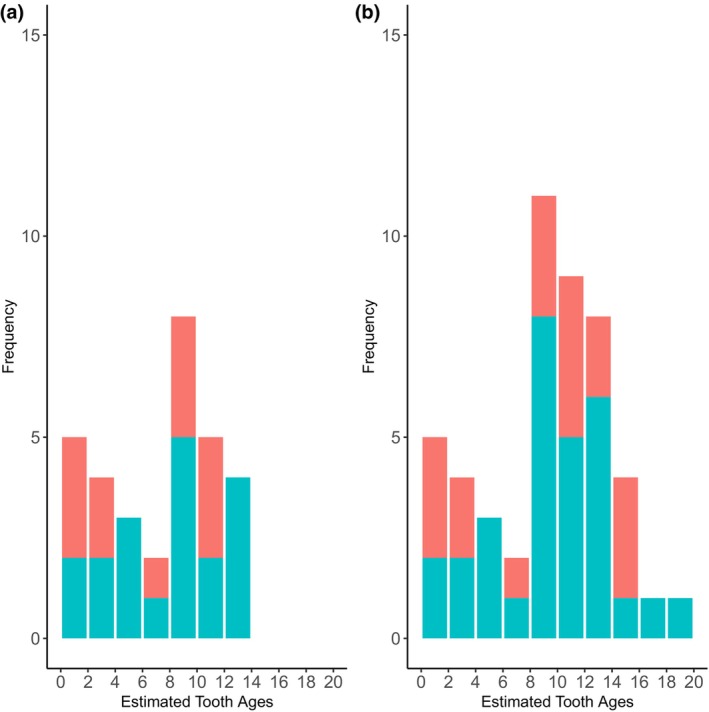
Age distributions of the Māui and Hector's dolphins in the (a) “strict” (*n* = 31) and (b) “relaxed” (*n* = 48) training sets. Blue bars are the number of females, and red bars are the number of males.

Paired teeth and skin samples were collected from 79 beachcast or bycaught Māui and Hector's dolphins by New Zealand Department of Conservation/Te Papa Atawhai, local Māori (Indigenous people of Aotearoa) or Massey University researchers between 2004 and 2017. The details about age estimates from tooth growth layer groups are provided in Betty et al. (2022). In brief, teeth were decalcified, sectioned, and growth layer groups were counted following the protocol of Slooten (1991) as modified by Duignan et al. (2004). Sections were counted by two readers, and a consensus age estimate was reached. Additionally, age estimates were assigned to one of four categories based on confidence in the age estimate and other tooth attributes, such as missing centroids or damaged growth layer groups. For the purposes of model building and performance, we selected two subsets from those provided in Betty et al. (2022): a “strict” subset (*n* = 31, Figure 1a) of individuals with high confidence in age estimates and a “relaxed” subset (*n* = 48, Figure 1b) of individuals with at least a minimum age estimate (Table S1).

Skin samples from live dolphins were collected by the University of Auckland/Waipapa Taumata Rau researchers or the Department of Conservation using a small, lightweight biopsy dart (PaxArms NZ Ltd., Cheviot, New Zealand) fired from a modified veterinary capture rifle. Calves of the year, defined as individuals approximately half the size or less of an adult and assumed to be less than 1 year old (Webster et al., 2010), were not sampled as part of these surveys. Biopsy samples were stored in 70%–90% ethanol at −20°C prior to DNA extraction. Additional details about survey methodologies are provided by Oremus et al. (2012) and Constantine et al. (2021).

### 2.2 DNA extraction and profiling

Genomic DNA was extracted from skin samples using a modified phenol–chloroform protocol for small samples (Baker et al., 1994). Extracted DNA was treated with RNAse A (1.4 μL of 1 mg/mL to samples of 140 μL for 30 min at room temperature) and purified using a Zymo PCR Inhibitor Removal Kit (Zymo Research Corp.). DNA concentrations were measured on a Qubit 4 fluorometer (Thermo Fisher). Sex was identified via a multiplex PCR of marker sets standard for Māui and Hector's dolphins (Hamner et al., 2017). PCR products were visualized by agarose gel electrophoresis to confirm sexes (for necropsied individuals) or assign sexes (for biopsy individuals). Subspecies for all samples were confirmed by amplification and sequencing of approximately 700 bp at the 5′ end of the mtDNA control region (Hamner et al., 2012). Sequences were trimmed to align to a 360 bp reference sequence for the diagnostic ‘G’ haplotype for Māui dolphins or one of the many Hector's dolphin haplotypes (Constantine et al., 2021; Hamner et al., 2012).

Details about individual identification of Māui and Hector's dolphins via a panel of microsatellites have been reported previously (Baker et al., 2013, 2016; Constantine et al., 2021; Oremus et al., 2012). In brief, individuals were genotyped at between 14 and 26 loci, depending on when samples were collected and genotyped. Each locus was amplified individually, then coloaded with up to five other loci amplified from the same individual for sizing. Each amplification included a negative control to detect contamination and up to seven internal control samples to standardize allele binning with previous genotyping runs and to estimate genotyping error. Microsatellite genotypes were compared within and across years using CERVUS 3.0.7 (Kalinowski et al., 2007). Samples with identical genotypes were considered resamples of the same individual.

Aliquots of genomic DNA were bisulfite converted using a Zymo EZ‐96 DNA Methylation‐Gold Kit (Zymo Inc.) at the Neurosciences Genomics Core Facility at the University of California Los Angeles, USA. When possible, 250 ng of genomic DNA was used for each individual. Methylation state of the genomic DNA was assessed using a custom Infinium mammalian methylation array with 37,491 oligonucleotide probes designed from mammalian genomes (HorvathMammalMethylChip40, Arneson et al., 2022). Unsupervised hierarchical clustering based on the interarray correlation with the R function *hclust()* was used to visually detect technical outliers (*n* = 8), which were then removed from further analysis. Fluorescence at the terminal nucleotide was read by an Illumina iScan machine and saved as idat files. Raw methylation data were normalized using the SeSAMe pipeline (Zhou et al., 2018), providing an estimate of methylation at each probe (Beta values, ranging from 0 to 1, with zero indicating no methylation occurred), and a *p*‐value for the confidence in the methylation estimate.

### 2.3 Quality control and molecular clock construction

Following Bors et al. (2021), *p*‐value filtering was applied to the methylation data using a custom R script. CpG sites with significant *p*‐values (<.05) in at least 10, 20, and 30 individuals were identified using the *p*‐values generated by the SeSAMe pipeline and counting these significant occurrences across individuals in the training subset. These subsets of CpGs were considered in model construction, along with the full, unfiltered set of CpGs. The Pearson's correlation coefficient was calculated to assess relationships between methylation values at individual CpG sites and age estimates from tooth growth layer groups.

Clock models were constructed using the *glmnet* package (Friedman et al., 2010) in R v 4.2.1 (R core team, 2022). The *glmnet* package allows for the construction of penalized multiple linear regression models by way of two parameters, lambda and alpha, that allow the elastic net model to vary between a ridge (α = 0) and lasso (α = 1) regression. Models were constructed for both subsets of calibration individuals and for the different CpG *p*‐value filtering subsets (e.g., no filtering, significant *p*‐values in 10 individuals, etc.) using the *cv.glmnet()* function for internal 10‐fold, cross‐validation that minimized values of lambda. The effect of alpha on candidate clock models was investigated by running model iterations for values of alpha between 0.1 and 0.9 in increments of 0.1. Model performance was evaluated by calculating Pearson's correlation coefficient between predicted model ages (using the *predict()* function in R) and tooth age estimates and by calculating absolute mean and median age errors. Model performance was further evaluated by running a leave‐one‐out cross‐validation (LOOCV) with the strict subset of calibration individuals. LOOCV models were also run with the CpG subsets and the same sequence of alpha values used during clock construction. As with the main set of clock models, LOOCV models were evaluated by calculating age errors and the correlation between tooth age estimates and predicted model ages for the individual removed in each LOOCV iteration.

We ran two separate logistic models with *cv.glmnet()* to investigate the potential confounding effects of subspecies and tissue sources on clock models. Specifically, we were concerned that most of the training individuals were dead beachcast Hector's dolphins, while most of the test individuals were derived from biopsy samples of live Māui dolphins. Subspecies and tissue sources were encoded in binary, where “1” indicated a Māui dolphin or biopsy tissue source and “0” indicated a Hector's dolphin or beachcast tissue source. Unlike the clock models described above where alpha was optimized for model performance, the parameter was instead set at 0.5 for these tests. The CpG sites selected for these models were inspected with the *match*() function in R to determine if any sites diagnostic of subspecies or tissue sources were included in candidate clock models.

To leverage existing methylation data and potentially improve age estimation for older Māui and Hector's dolphins, we also constructed a set of models that included skin methylation data from beluga whales collected in Cook Inlet, Alaska, USA (Bors et al., 2021; Horvath et al., 2021) following the conventions of similar multispecies clock models (e.g., Parsons et al., 2023; Robeck, Fei, Lu, et al., 2021b). The training individuals from Bors et al. (2021, *n* = 67) were included with both the strict and relaxed subsets of Māui and Hector's dolphins for merged training sets of 98 and 115 individuals, respectively. The beluga/dolphin clocks were constructed and evaluated as described previously for the species‐specific Māui and Hector's clocks. We used the *match()* function to check for overlap between CpG sites selected between the best‐performing models and the previously published beluga epigenetic clock (Bors et al., 2021) and the odontocete epigenetic aging clock (OEAC, Robeck, Fei, Lu, et al., 2021b).

The best‐performing species‐specific and joint models were used to estimate the DNAm ages for the remaining biopsy samples without a corresponding tooth sample (*n* = 135). Potential differences in clock distributions were assessed using a Wilcoxon‐signed rank test in R with the *wilcox. test()* function with paired = true. A subset of these samples are time points from individuals sampled years apart (*n* = 32), and thus we were able to evaluate model performance again by checking age prediction over time, though we did not attempt to optimize model performance further to refine these predicted sampling intervals.

### 2.4 Genomic location of model CpGs

CpG probe locations in the human or mouse genomes are known from methylation array design (Arneson et al., 2022). The genomic locations of the eight clock CpGs and flanking sequences (200 bp in both directions) were extracted from the human genome through the NCBI genome data viewer based on the RefSeq database (O'Leary et al., 2016). Extracted human sequences were then located in the bottlenose dolphin (*Tursiops truncatus*) genome (GCF_011762595.1) with NCBI BLAST (Altschul et al., 1990; Johnson et al., 2008), as this is the closest Māui dolphin relative with an annotated genome. The gene containing or located closest to the CpG site was recorded from the BLAST result. For those containing a CpG site, the molecular family, cellular component, and biological processes were noted by submitting the annotation name to GO Enrichment Analysis powered by the Panther tool at the Gene Ontology Resource (geneontology.org, Ashburner et al., 2000, Mi et al., 2019, The Gene Ontology Consortium, 2021).

### 2.5 Application of epigenetic age estimates to Māui dolphin age structure

DNAm ages were applied to investigate changes and the recent status of the Māui dolphin population (Constantine et al., 2021). Histograms of the age structure for all biopsy sampled dolphins (2001–2021) and during the 2015–2016 and 2020–2021 survey periods were generated to obtain a representation of age structure. Potential shifts in age distributions between the two periods were assessed using Wilcoxon rank sum tests in R with the *wilcox.test()* function.

## 3 RESULTS

### 3.1 Summary of training datasets

Tooth age estimates for the individuals in the strict subset of training individuals ranged between 0.25 and 14 years, with a median age of 8.5 years. This subset included 19 females and 12 males, for a sex ratio of 1.5 females to every male. In the relaxed subset, age estimates ranged between 0.25 and 20 years, with a median age estimate of 10 years. The sex ratio in this subset was 1.6 females to every male (*n* = 30 females and 18 males). Neither of the sex ratios were significantly different from a 1:1 expectation based on a binomial exact test (*p* > .05 for both). Demographic information about the beluga training data set is provided by Bors et al. (2021).

### 3.2 *p*‐Value filtering

*p*‐Value filtering of CpG sites initially removed 7859 sites that showed no significant relationship to tooth growth layer groups in at least 10 individuals. The number of sites removed continued to increase up to 15,924 CpG sites when required to have a significant relationship with most (*n* = 30) tooth‐aged individuals.

### 3.3 Single CpG correlations with known ages

The majority of retained CpG sites did not have a significant correlation coefficient with age in either calibration subset (Table 1). Pearson's correlation analyses with age for the strict subset of individuals found 4748 CpG sites with a significant age correlation, with 49.6% of sites having a positive correlation coefficient. The sites with the greatest positive and negative correlation coefficients were cg16496042 and cg20582188 with correlation coefficients of .848 and −.885, respectively. The same workflow with the relaxed set of individuals found 3500 CpG sites with a significant relationship to tooth age estimates, with sites cg16496042 and cg25254739 having correlation coefficients of .783 and −.787, respectively.

**TABLE 1:** Summary of absolute values of Pearson's correlation coefficients between tooth age estimates in the training set of dolphins and the methylation at CpG sites, including the percent positive in each bin.

Absolute value Pearson's *r*	Number of CpGs	% positive correlation
0–.1	9560	53.7
.1–.2	7754	53.7
.2–.3	5407	54.6
.3–.4	3642	57.8
.4–.5	1960	54.4
.5–.6	834	43.2
.6–.7	388	23.5
.7–.8	122	6.6
.8–.9	28	7.1
.9–1.0	0	NA
Total	29,695	53.5

### 3.4 Selection of molecular clocks

The elastic net regression provided a number of molecular clocks with high predictive accuracy for estimating DNAm ages of Māui and Hector's dolphins. Candidate species‐specific clocks varied in the number of terms from three to 112 CpG sites (four to 113 terms including the y‐intercept, Table S2). Generally, models constructed from the strict calibration dataset resulted in greater correlations between the estimated tooth age and DNAm age (strict correlation range: .93–.99, relaxed correlation range: .81–.94, Table S1) and had smaller age errors than those from the relaxed calibration dataset (strict error range: 0.09–1.77 years; relaxed error range: 1.59–2.49 years). As alpha increased, the number of terms in the model decreased; however, the number of terms in candidate models did not appreciably change as the threshold of significant *p*‐values in a subset of individuals increased beyond 10 individuals. Examination of the selected CpG sites in candidate models indicated that at smaller values of alpha extraneous sites were selected that sometimes improved model fit but over‐parametrized the model.

**FIGURE 2:**
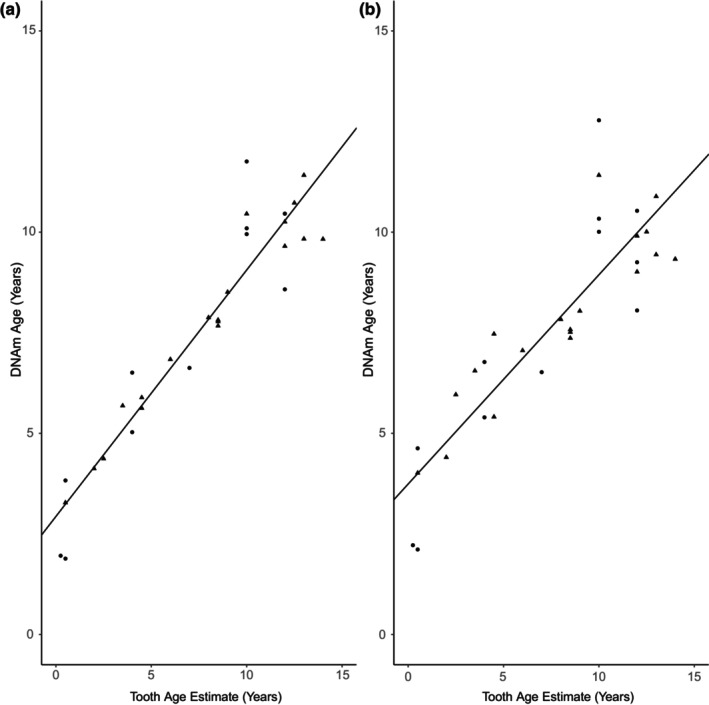
Scatterplots of the relationship between age estimated from tooth growth layer groups and from methylation data for the “strict” training set of Māui and Hector's dolphins for alpha = 0.9 for the (a) species‐specific clock model and (b) the leave‐one‐out cross‐validation analysis. The solid line is the modeled relationship between tooth age and DNAm age. Males are indicated by circles and females by triangles.

The final model selected as the epigenetic clock for Māui and Hector's dolphins originated from the strict model subset, contained eight CpG sites selected from those with significant *p*‐values in at least 10 individuals, had an age correlation of 0.95 and median absolute age error of 1.54 years (Table 2). This iteration was selected as it optimized model performance by minimizing age errors, avoided overparametrizing the relationship relative to other candidate models, and had a large correlation between the estimated tooth ages and the DNAm ages (Figure 2a). A LOOCV analysis with the strict calibration data set confirmed that this was the optimal choice (minimum 10 significant *p*‐values for CpG sites, α = 0.9, Table 3, Figure 2b) as this also optimized the age correlation and age errors relative to other CpG subsets and values of alpha (Table S3).

**TABLE 2:** Summary of the Māui/Hector's dolphin molecular clock based on the strict calibration subset.

Probe ID	Model coefficient	CpG correlation
(Intercept)	−4.6720053	NA
Cg00817637	0.2573007	.6955356
Cg09026530	−2.0929561	−.8658735
Cg09402653	2.4794632	.6847672
Cg09461098	−0.8055783	−.8669490
Cg16496042	2.7959477	.8480292
Cg20582188	−9.9140532	−.8854617
Cg24276148	16.2120942	.7576894
Cg25254739	−5.6220444	−.8444300

*Note*: CpG sites are referenced to the array probe name. Correlations are the Pearson's correlation coefficient between the estimated tooth ages and methylation at each probe.

**TABLE 3:** Statistics for the Māui/Hector's dolphin and joint beluga/dolphin epigenetic clock models and leave‐one‐out cross‐validation (LOOCV).

CpG subset	Alpha	Mae	Medae	*R* ^2^	*p*‐Value	Slope	y‐Intercept
Māui/Hector's dolphin clock							
10	0.9	1.59	1.54	.89	7.8e‐16	0.61	2.93
	LOOCV						
10	0.9	2.08	2.09	.76	1.4e‐10	0.52	3.74
Beluga/dolphin clock							
30	0.3	2.08	1.65	.94	<2.2e‐16	0.84	2.7
	LOOCV						
30	0.3	3.51	3.08	.81	<2.2e‐16	0.70	5.04

*Note*: Mean age error (mae), median age error (medae), *r* ^2^ for the regression, *p*‐value for the regression, regression slope, and y‐intercept for the regression.

The logistic penalized regression models to investigate potential confounding effects of sampling selected five CpG sites that were diagnostic of subspecies and 29 CpGs that were diagnostic of tissue source (Table S4). None of these CpG sites were included in any of the candidate clock models to estimate Māui dolphin age, nor in the final beluga/dolphin clock model.

The joint beluga/dolphin training sets produced clock models with more CpG sites, larger correlations, and comparable errors relative to the species‐specific Māui/Hector's clocks. Candidate model sizes ranged from 22 to 134 CpG sites, with correlations between tooth age and DNAm age estimates of .95–.97 (Table S5). The median age errors for these models ranged from 1.65 to 2.77 years. As with the species‐specific clock models, the use of the relaxed subset of dolphins generally decreased mean age correlations and increased age errors. The joint model that optimized the age correlation and minimized age errors had 77 CpG sites, an age correlation of .95, and a median age error of 1.65 years (Table 3, CpG sites and correlations in Table S6). The LOOCV analysis indicated that this model had an age correlation of .90 and a median age error of 3.08 years (Table 3; Table S7).

The best‐performing models had some CpG sites in common with the previously published beluga‐specific and OEAC models (Bors et al., 2021; Robeck, Fei, Lu, et al., 2021b). No sites were overlapping between the beluga‐specific and Māui/Hector's clock models (Table S8). Two CpG sites in the species‐specific Māui/Hector's clock overlapped with the OEAC. Unsurprisingly, 15 CpG sites in the beluga‐specific clock overlapped with the joint beluga/dolphin clock. One CpG site, cg15809488, was found in the OEAC, beluga‐specific, and joint beluga/dolphin clock.

### 3.5 Estimated age of living dolphins

**FIGURE 3:**
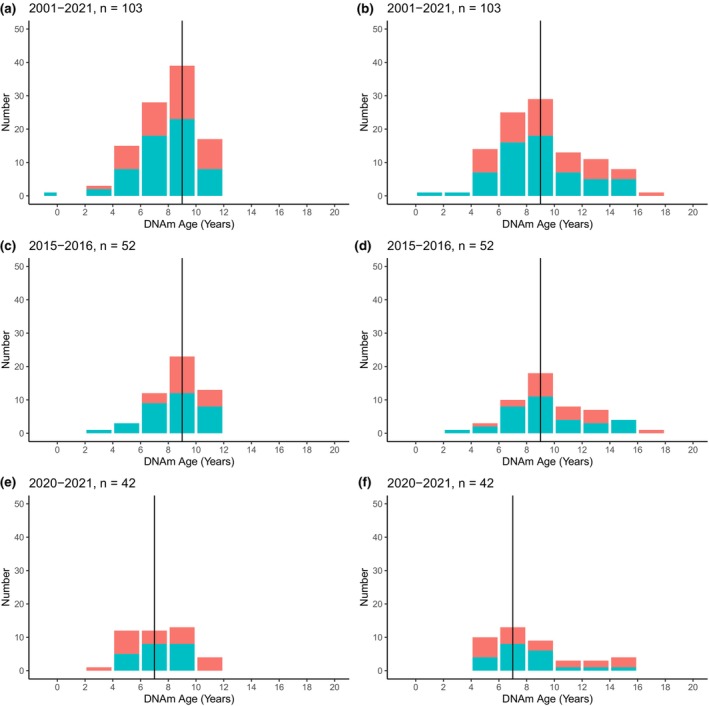
Age distributions for Māui dolphins estimated using the Māui/Hector's model (panels a, c, e) and the beluga/dolphin clock model (panels b, d, f). Panels a and b are the distribution for all sampled Māui dolphins not in the training data. Panels c and d are the distributions for individuals sampled during the 2015–2016 survey. Panels e and f are the distribution for the 2020–2021 survey. The solid vertical lines are the median ages, blue bars are the number of females, and red bars are the number of males. The youngest individual in panels a and b was a near‐term fetus that died in utero and thus would be expected to have an age less than 1.

Application of the two best‐performing models to the biopsy samples from living dolphins provided estimated ages between −0.5 and 21 years old (Figure S1). When restricted to just Māui dolphins, estimated ages with both clocks ranged between −0.5 and 17.3 years (Figure 3a,b, Table S9). The Māui/Hector's clock estimated an average age of 7.9 ± 2.1 years, with ages between −0.5 (for a near‐term fetus) and 11.7 years. The best‐performing beluga/dolphin clock estimated ages between 0.59 and 17.3, with an average of 9.1 ± 3.2 years. Median ages from the two clock models was significantly different (V = 691, *p* = 6.34e‐14, Māui/Hector's clock 95% confidence interval (CI) [7.61, 8.46]; beluga/dolphin clock 95%CI [8.28, 9.58]). The age intervals for serially sampled individuals were generally in the correct direction (e.g., older animals were older from samples 1 to 2), but intervals and absolute ages tended to be underestimated with both clock models.

### 3.6 Genomic location of model CpGs

Half of the eight CpG sites in the Māui/Hector's clock were located within genes found in the bottlenose dolphin genome (Table S10). The GO enrichment analysis indicated that these four CpG sites were associated with genes linked to protein or mRNA binding. These results should be considered preliminary and may change upon publication, annotation, and analysis of a Māui or Hector's dolphin genome. The other four CpG sites in the clock model were 2000–200,000 bp away from the closest annotated gene.

### 3.7 Māui dolphin age structure

Between the two survey periods of 2015–2016 and 2020–2021, the estimated age distribution of Māui dolphins shifted to younger individuals (Figure 3c–f). With the Māui/Hector's clock, the mean estimated dolphin age was 8.5 years in 2015–2016 (95% CI 8.18, 9.15) and decreased to 7.4 years in the 2020–2021 survey (95% CI 6.72, 8.05). Likewise, with the joint beluga/dolphin clock, the mean age between the two survey periods decreased from 9.7 to 8.5 years (95% CI 2015–2016: 8.74, 10.53, 95% CI 2020–2021: 7.26, 9.29). The estimated age distributions between the two periods were significantly different with both clock models (Māui/Hector's clock: W = 1476, *p* = .004, beluga/dolphin clock: W = 1357, *p* = .04).

## 4 DISCUSSION

The models presented here reveal the value of multi‐species clocks for a critically endangered species with limited sample sizes for age calibration. Clocks applicable to multiple species have also been developed for other odontocetes (Robeck, Fei, Lu, et al., 2021b) and long‐lived cetaceans (Parsons et al., 2023). Compared to these recent clocks, the models we developed have comparable or larger age correlations, comparable or smaller median age errors, and produced more realistic age estimates. For example, using the OEAC (Robeck, Fei, Lu, et al., 2021b), the training sets of Māui and Hector's dolphins had DNAm ages overestimated relative to estimated tooth ages and the species‐specific clock presented here (Hernandez et al., 2022, Table S11, Figure S2). This consistent overestimation could be a function of the species used in the OEAC training data, most of which have life spans comparable to, or decades longer than, those of Māui and Hector's dolphins (estimated longevity of 20 years, Slooten, 1991).

The accuracy and utility of epigenetic clock models are dependent on the available training information. In a review of best practices, Mayne et al. (2021) suggest a minimum training set of 70 individuals, with 134 or more being preferable for clock development. While such targets are possible with well‐studied species and populations, such as bottlenose dolphins in Sarasota Bay, USA or Shark Bay, West Australia (Beal et al., 2019; Peters et al., 2022), this is more difficult for less intensively studied, cryptic, or critically endangered species, particularly those with small estimated census sizes, such as Māui dolphins. In our case, the availability of samples from closely related Hector's dolphins proved critical for clock development. Further, we confirmed that CpG sites included in the clock models were not reflective of the different subspecies and thus would not bias age estimates. Toothwear may have impacted the known ages of the Māui and Hector's dolphins in our training data. Two individuals in the relaxed training subset were also known from genetic capture‐recapture and sighting histories before their deaths. Based on their sighting histories, both individuals were older and near the maximum expected age for Māui and Hector's dolphins (around 20 years), but tooth age estimates were 5–6 years younger than expected (Betty et al., 2022). While the Māui/Hector's clock calculated age estimates similar to the estimated tooth ages, the joint beluga/dolphin clock estimates were more consistent with those from sighting histories and genetic capture‐recapture. This further supports the recommendation to include individuals across the range of potential ages in the training dataset (Mayne et al., 2021). In our case, while we had good representation across the youngest and middle ages for Māui and Hector's dolphins, there were few older individuals available for inclusion in the training dataset. Continued sampling of beachcast and live Māui and Hector's dolphins can help to further refine clock performance.

The current population of Māui dolphins is estimated at 54 individuals aged 1 year or older (Constantine et al., 2021; Hamner et al., 2014) and may decline further (Cooke et al., 2019). There is a lack of knowledge on basic demographic parameters such as age at sexual maturity and reproductive rates, with inferences made from Hector's dolphin research (Slooten, 1991; Slooten & Lad, 1991). This may have limitations when applied to the highly range‐restricted, genetically isolated Māui dolphin population. Critically endangered populations are often under selective pressures, including Allee effects associated with genetics, social structure, reproduction and predation, or interactions between multiple effects (e.g., Berec et al., 2007; Frère, Krützen, Mann, et al., 2010a; Gascoigne et al., 2009; Stephens & Sutherland, 1999). For highly social species like dolphins, it is important to know the age of sexual and social maturity, that is, the age at first reproduction, as these can differ by several years, and whether there is variation in reproductive success by individual and/or age class (Frère, Krützen, Kopps, et al., 2010b; Henderson et al., 2014). Younger animals also may not yet have learned the social cues or behaviors necessary to successfully reproduce.

An important indicator of potential population recovery is the age‐curve which when skewed to the left indicates greater recruitment through survival of offspring. Here we found that the mean and median ages of Māui dolphins have decreased across two recent surveys conducted 5 years apart (Figure 3c–f). This may be a consequence of the establishment of the West Coast North Island Marine Mammal Sanctuary in 2008, which largely removed the primary threat—the risk of bycatch. With an estimated generation time of 13 years (Taylor et al., 2007), individuals born after 2008 would have a greater chance of survival as would their offspring. In addition, the exclusion of fishing pressure may have resulted in greater prey availability through ecosystem change (Ogilvy et al., 2022). While it is not yet known what impacts this younger population may have on the persistence of the Māui dolphins, one unintended benefit of a younger population could be increased disease resistance (Beineke et al., 2010; Tejada‐Martinez et al., 2021). While the removal of toxoplasmosis as a threat would be an ideal solution for Māui dolphin persistence, some low level of exposure to the oocytes could potentially confer some degree of resistance that then incorporates itself into subsequent generations. Continued monitoring of the population could reveal changes in their life history. Boat‐based surveys and genetic capture‐recapture have suggested an increasing occurrence of young dolphins in the population, while older individuals are not being encountered, possibly because they are not living to the maximum expected age (Constantine et al., 2021). It remains to be seen if this high turnover in individuals is related to more younger individuals recruiting into the population or a continued loss of older adults.

The models presented here demonstrate how to overcome sample limitations when developing epigenetic clocks for species of conservation concern that may lack adequate tissue archives for training data. Additionally, this could prove useful for cryptic species where encounters are rare, but age estimates could be useful for an improved understanding of population structure. For example, a beaked whale (Family Ziphiidae) clock could be developed from frequently encountered species (e.g., Cuvier's or Blainville's beaked whales) and applied to less frequently encountered members of the family from biopsy samples. The age estimates obtained here will help to contextualize subsequent demographic studies on Māui and Hector's dolphins, including kinship analyses, as well as prove useful to conservation managers.

## AUTHOR CONTRIBUTIONS

**Keith M. Hernandez:** Conceptualization (equal); data curation (lead); formal analysis (lead); methodology (equal); software (lead); validation (equal); visualization (lead); writing – original draft (lead); writing – review and editing (equal). **Kaimyn B. O'Neill:** Conceptualization (equal); formal analysis (supporting); investigation (equal); writing – review and editing (equal). **Eleanor K. Bors:** Formal analysis (supporting); investigation (equal); methodology (supporting); software (supporting); writing – review and editing (equal). **Debbie Steel:** Conceptualization (equal); data curation (equal); investigation (equal); methodology (equal); writing – review and editing (equal). **Joseph A. Zoller:** Formal analysis (supporting); investigation (equal); software (equal); writing – review and editing (equal). **Rochelle Constantine:** Conceptualization (equal); data curation (equal); funding acquisition (equal); investigation (equal); project administration (equal); resources (equal); writing – review and editing (equal). **Steve Horvath:** Conceptualization (equal); data curation (equal); methodology (equal); project administration (equal); resources (equal); supervision (equal); validation (equal); writing – review and editing (equal). **C. Scott Baker:** Conceptualization (equal); funding acquisition (equal); project administration (equal); resources (equal); supervision (equal); validation (supporting); writing – review and editing (equal).

## CONFLICT OF INTEREST STATEMENT

SH is a founder of the nonprofit Epigenetic Clock Development Foundation which plans to license several of his patents from his employer UC Regents. The other authors declare no conflict of interest.

## BENEFIT‐SHARING STATEMENT

Dolphin samples were collected with the permissions and in collaboration with mana whenua (local Māori who are kaitiaki/guardians of the Māui dolphins) and this has been included in the Methods and Acknowledgments. Methylation data and R code will be archived in public repositories. Preliminary work from this project has been shared at a public forum on Māui and Hector's dolphin research in October 2022.

## Supporting information


Figure S1.

**Table S1.** **–S11.**

## Data Availability

The manifest for the methylation array (HorvathMammalMethylChip40) is available at the Gene Expression Omnibus (GPL28271: Illumina HorvathMammalianMethylChip40 Bead Chip). Māui and Hector's dolphin methylation data are partially archived in the NCBI GEO database at GSE164465, “Genome Methylation in Wild Beluga Whales” (Accession numbers GSM5011491–GSM5011500), and remaining methylation data are archived at GSE242072, “Epigenetic aging of Māui and Hector's Dolphins” (Accession numbers GSM7748508–GSM7748648) and will be made public upon manuscript acceptance (private link for peer review: ovqhwoccxrajpgf). Beluga methylation data are archived in the NCBI GEO database, GSE164465, “Genome Methylation in Wild Beluga Whales.” R code and accompanying files are archived in Dryad upon acceptance: https://doi.org/10.5061/dryad.hx3ffbgm3. The mammalian methylation array is available from the nonprofit Epigenetic Clock Development Foundation.
