# Supplementary material for: Using epigenetic clocks to investigate changes in the age structure of critically endangered Māui dolphins
Source: Ecol Evol. 2023 Sep 28;13(10):e10562. doi: 10.1002/ece3.10562 (PMC10534197; doi:10.1002/ece3.10562)
Supplement: Supplementary file 1 — Figure S1. [file ECE3-13-e10562-s002.docx]

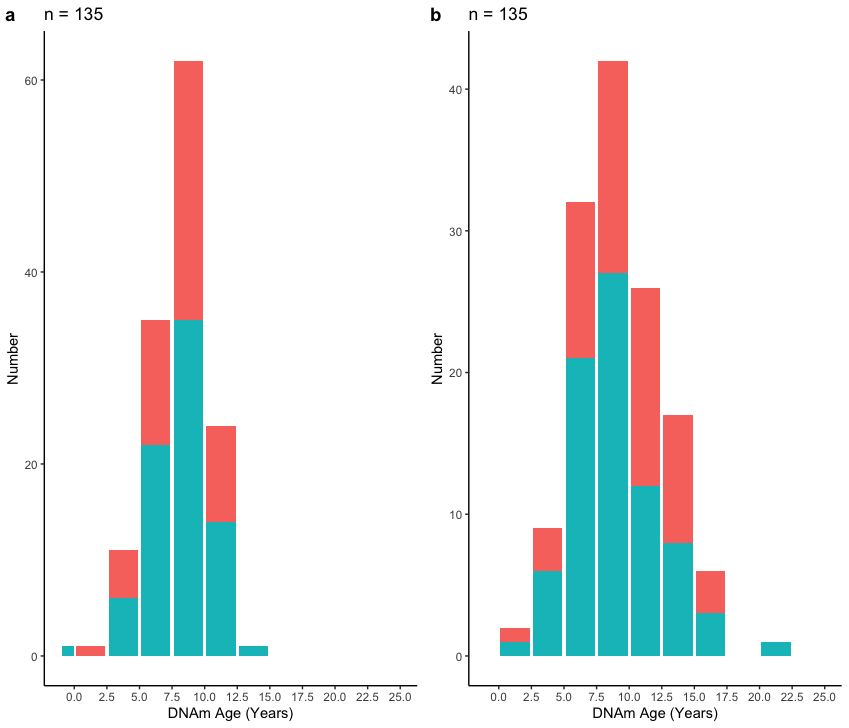


Figure S1: Age distributions for Māui and Hector’s dolphins estimated using the Māui/Hector’s (a) and beluga/dolphin (b) clock models. Red bars are the number of males and blue are the number of females. The youngest individual in both plots is a near-term fetus that diet *in utero* and would thus have an expected age less than 1.


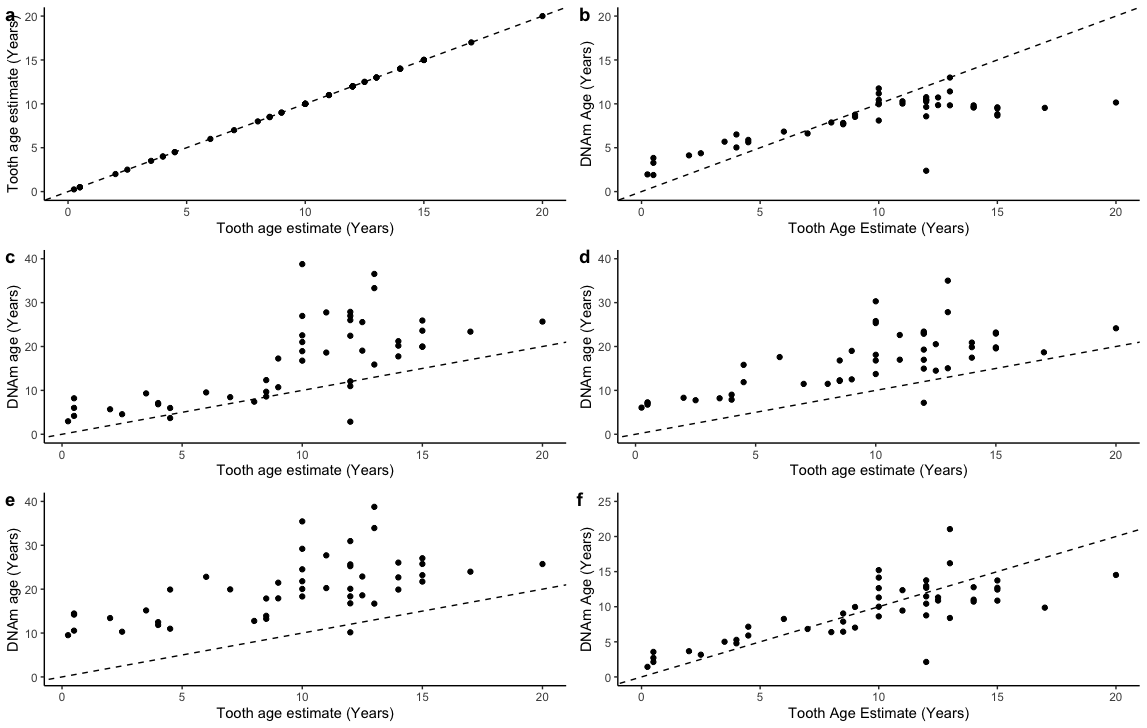


Figure S2: Scatterplots of the relationships between estimated tooth ages of Māui and Hector’s dolphins in the relaxed training set (n = 48 individuals) relative to DNAm ages produced by different epigenetic clock models. In all plots, individuals are indicated by circles and a 1:1 reference line is indicated by a dashed line. (a) A regression of tooth ages against tooth ages for reference. (b) Estimates from the Māui/Hector’s specific clock (this study), (c) estimates from the odontocete epigenetic aging clock (OEAC, Robeck et al. 2021b), (d) estimates from the beluga-specific clock (Bors et al. 2021), (e) estimates from a killer whale-specific clock (Parsons et al. 2023), and (f) estimates from the beluga/dolphin clock (this study).

**Supplementary Table Captions**

Table S1: Demographic and subset information for the training subsets of Māui and Hector’s dolphins. Ages are the tooth age estimates provided by the Department of Conservation – Te Papa Atawhai.

Table S2: Model iteration statistics for both the strict and relaxed model subsets. nCpGs = number of CpG sites in a model iteration; AgeCorr is the Pearson’s correlation coefficient between tooth age estimates and DNAm ages; R2 is the R^2^ of the modeled relationship between tooth age estimates and DNAm age; Medae is the median absolute age error; Yint is the y-intercept of the modeled relationship; RegSlope is the slope of the regression between tooth age estimates and DNAm age.

Table S3: Leave-one-out cross-validation (LOOCV) statistics for the strict training subset of models. Refer to Table S2 and the Read Me tab for abbreviations.

Table S4: CpG sites and coefficients for potentially confounding sites associated with tissue source and subspecies.

Table S5: Model iteration statistics for the strict and relaxed subsets of beluga/dolphin clocks. Refer to Table S2 and the Read Me for abbreviations.

Table S6: CpG sites, model coefficients and Pearson’s correlations for the final beluga/dolphin clock model.

Table S7: Leave-one-out cross-validation (LOOCV) statistics for the strict and relaxed model subsets for the beluga/dolphin clocks. Refer to Table S2 and the Read Me tab for abbreviations.

Table S8: Overlapping CpG sites between the Māui/Hector’s, beluga/dolphin, beluga-specific and odontocete epigenetic aging clocks (OEAC).

Table S9: Predicted DNAm ages for the test set of Māui dolphins (n = 103). MH prediction is the DNAm age prediction from the Māui/Hector’s clock, and BD prediction is the predicted DNAm age from the beluga/dolphin clock.

Table S10: Gene Ontology (GO) database annotations for the eight CpG sites in the Māui/Hector’s clock. Human genome annotations are from the HorvathMammalMethylChip40 array manifest. Refer to the Read Me tab for abbreviations.

Table S11: Predicted DNAm ages for the training set of Māui and Hector’s dolphins based on the odontocete epigenetic aging clock (OEAC, Robeck et al. 2021b), the beluga-specific clock (Bors et al. 2021), a killer whale clock (Parsons et al. 2023), the Māui/Hector’s and beluga/dolphin clock (this study). DOC_age is the estimated tooth age provided by the Department of Conservation – Te Papa Atawhai.
